# Precision oncology at speed: workflow design determines clinical utility in rapid molecular testing for lung cancer

**DOI:** 10.1093/oncolo/oyag388

**Published:** 2026-10-01

**Authors:** Brandon S Sheffield, Katherina Baranova, Parneet Cheema, Miriam A Bredella, Champica Nicholas, Ibiayi Dagogo-Jack, Jochen K Lennerz

**Affiliations:** Division of Advanced Diagnostics, William Osler Health System, Brampton, Ontario, L6R 3J7, Canada; Schulich School of Medicine and Dentistry, London, Ontario, N6G 2V4, Canada; University of Toronto, Toronto, Ontario, M5S 1A1, Canada; NYU Langone Health, Grossman School of Medicine, New York, NY 10016, United States; Division of Advanced Diagnostics, William Osler Health System, Brampton, Ontario, L6R 3J7, Canada; Massachusetts General Hospital, Cancer Center, Boston, MA 02114, United States; Natera, Austin, Texas, 78753, United States

**Keywords:** rapid testing, lung cancer, biomarker, molecular testing

## Abstract

**Purpose:**

Matching patients with effective targeted therapies requires timely molecular results; however, the challenge today how to embed it into routine clinical workflows in ways that meaningfully affect patient-level treatment decisions.

**Methods:**

Building on prior work, the authors systematically reviewed laboratory operations, interdepartmental coordination, and administrative processes from two distinct healthcare settings: a US-based academic tertiary care center and a Canadian community hospital. We identified shared themes, context-specific challenges, and operational elements that distinguished successful integration from assay availability alone.

**Results:**

Across both environments, the comparison revealed that rapid testing consistently accelerated treatment decisions and facilitated biomarker-informed patient management, but its successful implementation depended on re-engineering laboratory workflows, aligning personnel competencies, and establishing turnaround time monitoring; technical availability alone did not ensure clinically meaningful rapid testing. While the academic context integrated rapid testing within comprehensive molecular pipelines and research frameworks, the community hospital context focused on pragmatic adoption for frontline care, especially in urgent clinical settings and based on the organization of the Canadian health system.

**Conclusion:**

Implementing rapid molecular testing in NSCLC is fundamentally an implementation and systems-engineering challenge rather than a technical challenge. Clinical speed depended more on system design than assay availability alone and is influenced, in part, by the larger national healthcare context. Addressing laboratory, operational, and administrative barriers is essential to ensure that rapid testing improves outcomes. Lessons learned from both academic and community practice provide a practical, implementation-focused roadmap for broader adoption and equity in access to precision oncology.

Implications for practice Comparing academic and community environments demonstrated that clinical speed depended more on workflow integration and operational design than assay availability alone. Across both settings, successful integration depended on workflow redesign, defined operational responsibilities, interdepartmental coordination, and real-time turnaround-time monitoring, enabling patients to receive actionable biomarker results early enough to consistently guide initial treatment decisions. Any size practice can use this article as a practical guide toward implementing and reenvisioning rapid molecular testing at their own institution.

## Introduction

Precision oncology has transformed the management of non–small cell lung cancer (NSCLC).^1–3^ Numerous gene alterations have been identified that impact therapy selection, and testing of lung cancer specimens is recommended for identification of potentially efficacious targeted therapies, as well as avoidance of therapies unlikely to provide benefit.^4^^,^^5^ The ability to match patients with effective therapies now hinges on timely access to reliable molecular testing results, placing the molecular diagnostics laboratory at the center of precision oncology care for lung cancer.^6^

The emergence of biomarker-informed treatment as a standard of care in NSCLC, coupled with the aggressive nature of the disease, has created an urgent demand for rapid molecular testing.^7^ Workflows for same-day test ordering and reporting and even same-day treatment initiation are technically feasible and increasingly demonstrated in practice.^7–9^ However, the specific factors needed to realize broader access to rapid biomarker testing strategies are not readily available.^10^^,^^11^ In other words, the current challenge has shifted from technical capability to an implementation question: namely, how to embed rapid testing effectively into routine care.

Despite guideline inclusion, access to molecular testing remains inconsistent.^12–15^ Many patients with advanced NSCLC still do not undergo adequate testing,^16^^,^^17^ in part because comprehensive approaches such as next-generation sequencing require days to weeks to complete.^10^ Faced with clinical urgency, patients may begin empiric or non-matched therapy, a strategy that has been associated with worse outcomes when compared with biomarker-informed treatment outcomes.^18^^,^^19^ Recent studies further characterize this implementation gap. Suboptimal use of recommended panel testing and test-concordant therapy has been associated with life-years lost in patients with metastatic NSCLC,^20^ while analysis of 271 574 solid-tumor comprehensive genomic profiling tests identified that belays between biopsy and test ordering were the largest contributor to prolonged turnaround time.^21^ Together, these studies define important gaps in biomarker testing delivery but provide limited guidance on how healthcare systems can address them.

To provide practical context for addressing these implementation gaps, and based on the shared yet distinct experiences of successfully implementing rapid testing in different healthcare environments, the authors set out to compare and contrast factors that enabled sustained rapid test implementation in clinical care. Our aim is to highlight both common and setting-specific lessons. More broadly, this work underscores that clinical impact depends on thoughtful implementation. In this context, the molecular pathology laboratory plays a central role in how healthcare systems operationalize precision oncology.

## Methods

This review builds on prior work examining the implementation of rapid molecular testing in NSCLC within different healthcare contexts.^22–24^ Drawing from these experiences, both authors systematically reviewed, compared, and contrasted their respective practices. Dagogo-Jack and Lennerz describe the implementation of coordinated molecular testing and integrated diagnostics for NSCLC and other solid tumors at Massachusetts General Hospital (MGH), part of the private, nonprofit Mass General Brigham (MGB) healthcare system, within the United States’ mixed public-private, multipayer healthcare system. Sheffield, Baranova, Cheema, and Nicholas describe the implementation of rapid biomarker testing for NSCLC and other solid tumors in a community setting in a public, Canadian healthcare context. This work was exempt from institutional REB review as a Quality Improvement/Assurance Project.

MGH is a tertiary care hospital and academic medical center in Boston, Massachusetts, United States of America, with a subspecialized thoracic oncology practice, interventional radiology, and extensive participation in early- and late-phase clinical trials. The hospital’s Center for Integrated Diagnostics and affiliated pathology and oncology services support high-volume biomarker testing and novel precision-medicine workflows across multiple disease sites. The William Osler Health System (WOHS) is a multisite community healthcare organization with centralized lab services in Brampton, Canada. The laboratory serves a large medical oncology practice, including a subspecialized thoracic oncology practice, and supports a large number of phase 2 and 3 clinical trials. WOHS is situated in Ontario, Canada, where biomarker testing is centralized at designated testing centers for each disease site.

The analysis proceeded in two stages. First, we systematically identified clinical, operational, and regulatory drivers influencing the adoption of rapid biomarker testing in each healthcare setting. Second, we performed a structured comparison of laboratory workflows, operational models, and administrative frameworks across settings to delineate shared elements and context-specific differences. We further reviewed published literature for evidence in other contexts to support our findings. This approach generated (a) a consolidated assessment of practice-tested, practice-related benefits and challenges associated with rapid testing in NSCLC and (b) an implementation-focused comparison identifying laboratory, operational, and administrative aspects most relevant to adoption across diverse care environments.

## Results

Both centers implemented rapid in-house molecular testing in response to prolonged turnaround times and delayed access to biomarker-guided therapy. In both settings, clinical urgency was the primary driver for implementation. Prior to the introduction of rapid in-house molecular testing, both centers identified significant challenges and pressures that directly affected patient-level treatment timelines. In our context, CAP/IASLC/AMP guidelines recommend molecular testing for key oncogenic drivers and further specify a 10-day turnaround time metric between sample receipt and reporting of all results.^25^

Prior to implementation of in-house biomarker testing, in 2017, WOHS measured a median turnaround time for NSCLC biomarkers of 64 days (*n* = 99, IQR 31-100 days).^26^ This local context was impacted by both internal factors, including delays in initiation of testing and transport time, as well as external factors, including specimen backlogs at the centralized testing site. During this period (and in both settings), delays in biomarker reporting frequently extended beyond clinically meaningful decision windows, limiting the ability to initiate targeted therapy at first presentation (Figure 1). Medical oncologists relied on access to US-based private laboratory testing via clinical trials, private pay, or other promotions to gain access to appropriate biomarker testing for advanced NSCLC patients. Oncologists reported uncertainty with regard to access to biomarker testing and thus, targeted therapy.

**Figure 1.**
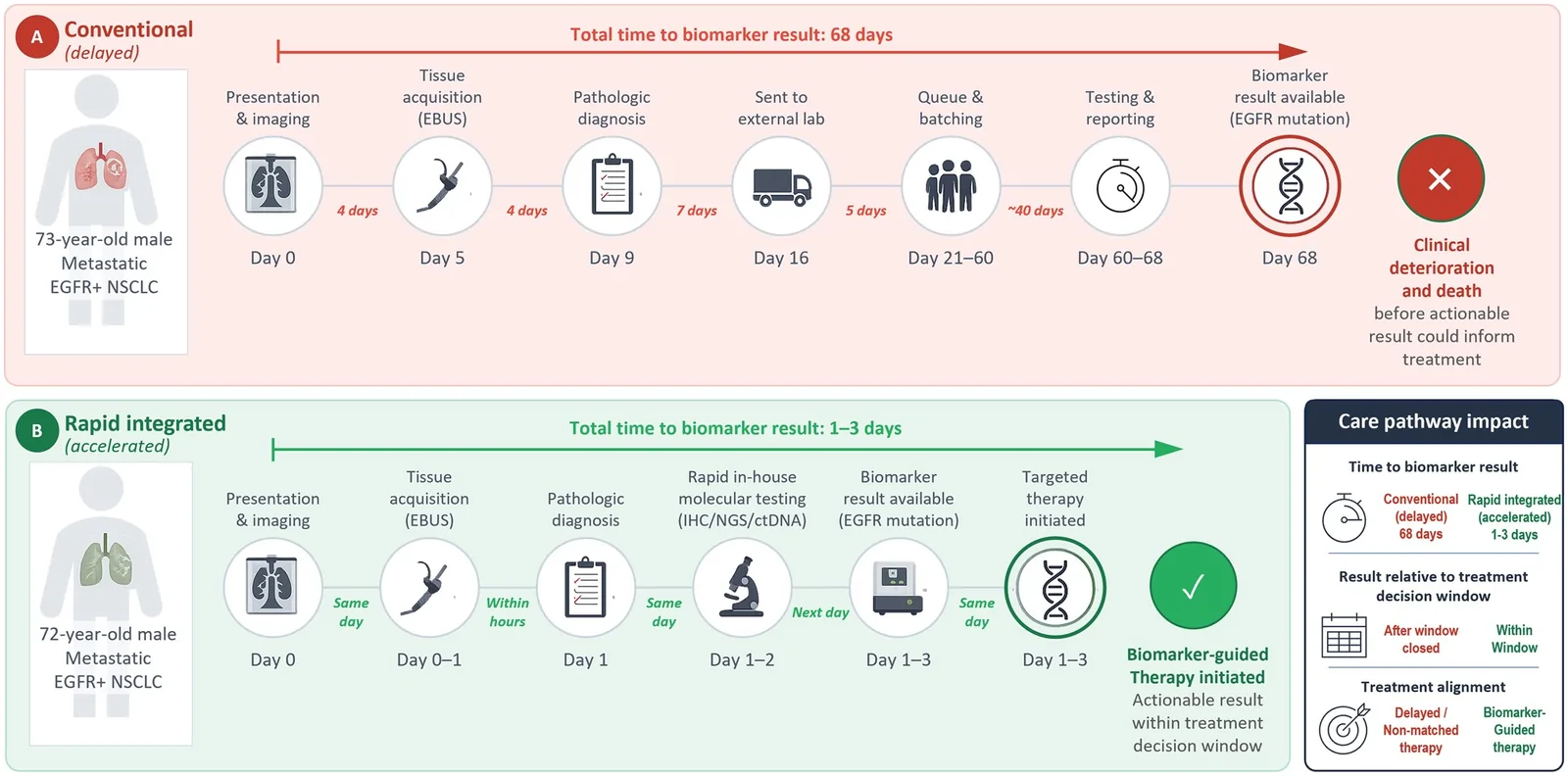
Diagnostic latency shapes time to actionable therapy in advanced non–small cell lung cancer (NSCLC). Illustrative comparison of two clinically similar patients with metastatic EGFR-mutated NSCLC demonstrating the impact of diagnostic workflow design on biomarker availability and treatment opportunity. (A) The upper panel depicts a conventional send-out testing pathway characterized by sequential ordering, specimen transport, batching, and external laboratory processing, resulting in prolonged turnaround time and delayed molecular result availability beyond the clinically relevant treatment window. (B) The lower panel depicts an integrated rapid-testing pathway using reflex molecular testing, in-house workflow integration, automation, and coordinated multidisciplinary communication to achieve biomarker availability within 1-3 days and enable biomarker-guided therapy initiation during first-line treatment decision-making. Diagnostic turnaround time is determined not only by assay performance but also by system-level operational factors, including ordering practices, transport logistics, laboratory integration, workflow coordination, and procedural integration. Timelines are illustrative of real-world institutional workflow variation.

Canadian centers continue to report substantial variability in NSCLC testing^27^ with turnaround times recorded in published data in the range of 5 to 86 days (mean: 24 days), with many studies reporting delays exceeding clinically actionable windows.^7^^,^^27–31^ Biomarker availability at time of oncologist appointment is a further metric which is critical to patient care. At WOHS, prior to implementation, only 30% of patients (*n* = 30/99) had a complete biomarker report available at time of oncology consultation.^26^ In another study, implementation of in-house biomarker testing increased available biomarker test results at their initial medical oncology consultation from 52% with send-out testing to 88%.^31^ A reduction in transit-time of several days and improvement in efficiencies can account for the improvement in terms of in-house compared to send-out testing. At WOHS, prior to implementation, delays internally in sending tissue to the external site was measured at up to two weeks in some cases.

MGH relied on a mix of internal and external testing for advanced biomarker assays, which resulted in prolonged turnaround times and variable access to emerging targeted therapies.^32^ Similar large academic centers in the United States continue to face comparable challenges in balancing centralized testing with rapid access to results for patients with advanced NSCLC.^16^ Post-implementation, consecutive improvement initiatives streamlined in-house molecular testing and data integration substantially, reduced turnaround times, and allowed real-time incorporation of biomarker results into clinical decision-making to ultimately achieve turnaround times from ordering to receipt of targeted treatment the same day.^23^

WOHS implemented in-house testing in 2019, associated with a median turnaround time of three days.^33^ In 2025-2026, the median turnaround time was three days for solid tumors, with specimen volumes of 1385 that year for NSCLC (internal data). For liquid biopsy, the mean TAT was measured at four days for 2025-2026 with specimen volumes of 216 for NSCLC (internal data). Solid tumor biomarker testing at WOHS was implemented within the immunohistochemistry (IHC) service, creating a new *advanced diagnostics* division.

Molecular testing is ordered reflexively, at the same time as IHC, therefore beginning the process of NGS testing concurrently with IHC-based biomarkers. Reflex workflows enabled concurrent IHC and NGS processing beginning on day 1, minimizing batching and sequential delays. Automation of extraction, sequencing, and analysis enabled next-day molecular results in many cases.^24^^,^^34–36^ In studies, integrated NGS results can be available in 24 hours.^35^^,^^37^ Nucleic acid extraction begins immediately after slide cutting (day 1), and sequencing typically occurs same day with results available next day (day 2). At WOHS, reporting is performed by a dedicated genome analyst, and the final integrated report signed out by a pathologist (days 2-3). NSCLC testing is also prioritized compared to other samples, although overall turnaround time across all samples is maintained under a week.

When comparing an academic tertiary center with a community hospital, distinct patterns of implementation emerged (Table 1). The academic setting leveraged rapid testing within a broader innovation ecosystem, embedding assays into complex molecular workflows, clinical trials, and regulatory frameworks with higher validation demands. In contrast, the community setting emphasized pragmatic integration into frontline care, prioritizing rapid triage and immediate treatment access within tighter resource constraints. This is reflected in the technologies utilized. Community hospitals have smaller volumes as well as fewer staff, and the technologies chosen for implementation at WOHS were selected for automation and ease-of-use, rather than broader panels or testing availability. Additionally, internal and external resource constraints impacted implementation and the initial turnaround time. Although centralization is required for centers with insufficient volumes or expertise to perform the necessary testing, in a resource limited context. Sending out cases can introduce delays across tissue procurement, specimen preparation, and transport, compounded by workforce and infrastructure limitations. In-house testing has been implemented in a variety of settings to improve turnaround-time, as well as other key metrics such as availability of test results at oncology consultation,^31^ utilization of biomarker guided therapy,^38^ and time to treatment.^39^^,^^40^ Together, these contrasting settings illustrate that implementation strategy is shaped more by clinical context, infrastructure, and workflow constraints than by assay selection alone. Across both settings, establishing and tracking turnaround time baselines before implementing rapid testing was essential, as it provided an objective measure of value gained through optimization and demonstrated the clinical impact of faster results.

**Table 1.** Implementation perspective: academic vs community.

Factor	Academic Tertiary Center (MGH)	Community Hospital (William Osler)
**Regulatory framework**	CLIA/CAP standards, emphasis on lab-developed tests and innovation	Health Canada & Provincial standards, focus on lab-developed biomarker tests over companion-diagnostic assays
**Clinical driver**	Need for comprehensive, actionable molecular results for advanced therapy selection	Need for rapid triage (ED/inpatient), focus on streamlining access and turnaround
**Infrastructure**	Large, subspecialized molecular lab; high testing capacity; integration with academic research	General pathology lab with broader but less specialized scope; limited molecular infrastructure. No dedicated research facilities
**Workflow integration**	Direct integration with tumor boards, research protocols, adaptive trial enrollment	Closer link to frontline care (oncology, ED), focus on immediate patient management
**Implementation barriers**	Regulatory complexity, balancing speed with comprehensiveness, higher validation burden	Limited infrastructure, resource constraints, reimbursement, and funding issues
**Opportunities**	Leadership in developing/validating new rapid tests; shaping policy and reimbursement	Using rapid panels to reduce inequities in access; pragmatic adoption in real-world community care

The overarching benefits and challenges observed across both settings are summarized in Table 2, highlighting the clinical, operational, patient-centered, and system-level tradeoffs associated with rapid testing. These dimensions frame rapid biomarker testing clearly as both a clinical accelerator and an organizational challenge. Rapid expansion can lead to shortcomings in other areas of the laboratory, and therefore optimization of every step of the process is critical.^41^

**Table 2.** Potential benefits vs challenges of rapid testing in NSCLC.

Dimension	Potential benefits of rapid testing in NSCLC	Potential barriers
**Clinical impact**	Faster turnaround → earlier therapy initiation; improved outcomes; better triage (ED/inpatient)	Risk of false negatives/positives if rapid but less comprehensive; clinical uptake and provider education needed
**Operational efficiency**	Reduced delays in care pathway; streamlined workflow; improved patient throughput	Need for infrastructure changes; workflow integration challenges
**Patient experience**	Shorter waiting times; less uncertainty; earlier relief (knowing treatment plan)	Risk of overpromising if not all actionable results available rapidly; Patients do not have a comparator and may not perceived incremental workflow improvements directly
**Health system**	Better resource allocation: reduced hospital stay costs; supports guideline adherence	Reimbursement gaps; sustainability of funding models; need for a coherent vision and interdepartmental alignment
**Equity**	Potential to democratize access (esp. in community hospitals with rapid panels)	Risk of widening disparities if only well-funded centers can implement
**Scientific/Innovation clinical trial relevance**	Real-world data generation; supports adaptive clinical trial enrollment	Limited evidence base for some rapid panels; regulatory uncertainty

### Laboratory operations

Workflow redesign across the diagnostic pathway emerged as the dominant determinant of rapid testing performance at both centers. In the academic setting, a subspecialized molecular service embedded rapid testing within complex existing workflows, requiring coordinated engagement across accessioning, analysis, and reporting to identify bottlenecks and prevent process duplication. Ongoing competency assessment and personnel monitoring were essential, given the technical and procedural demands of rapid testing. In the community setting, where resources are leaner and staff are more generalist, targeted panels provided a practical solution. At WOHS, workflows were optimized for speed while minimizing additional burden on personnel, space, and funding. Although the absence of a prior in-house biomarker infrastructure initially posed a barrier, it also enabled de novo adoption of streamlined rapid testing workflows rather than incremental modification of legacy processes.

### Interdepartmental coordination

Rapid testing depended on coordination beyond the laboratory itself. In tertiary academic centers, test results are closely tied to tumor boards, multidisciplinary care pathways, and clinical trial enrollment, which necessitate highly coordinated communication across oncology, pathology, and research teams. The community hospital perspective is anchored more directly in real-time patient management, especially in emergency and inpatient contexts, where coordination with oncology and internal medicine teams ensures that rapid results translate into timely therapeutic action. Tumor boards play a role in enhancing implementation as well as patient care in both settings, although the availability and level of engagement with tumor boards varies across settings. Even in resource-limited settings, multidisciplinary communication can be a metric with targets for improvement to help drive overall rates of testing.^42^ Concrete examples of how interdepartmental coordination enabled rapid testing across the diagnostic lifecycle are shown in Table 3. These examples span ordering, validation, pre-analytical, and post-analytical phases and illustrate how structured clinical–laboratory interaction translated assay availability into reliable, timely, and actionable results. Particularly, attention has to be paid to all steps of the workflow. When tissue is collected, it is vital that sufficient sampling is performed to optimize downstream adequacy for testing, which requires collaboration with interventional radiology.^43^ At the time of ordering, pathologist-initiated reflex testing can serve as a mechanism to decrease turnaround time and time to treatment.^28^^,^^44^ When a sample is suboptimal, this should be flagged, and plasma-based testing can be considered.^45^ Post-analytically, molecular tumor boards can serve as a mechanism to optimize actionability and interpretability of results.^38^

**Table 3.** Practical examples of clinical–laboratory coordination supporting rapid biomarker testing.

Implementation stage	Example of interdepartmental collaboration	Practical impact
**Ordering**	Oncologists alerted the laboratory when clinical features (e.g., never-smoker NSCLC) were discordant with negative molecular results, prompting reassessment or additional testing.	Improved quality control and reduced risk of missed actionable alterations.
**Validation**	Medical oncologists participated in assay validation by identifying and curating cases with rare alterations (e.g., RET, NRG1 fusions) from clinical trial and comprehensive testing cohorts.	Strengthened validation of rare but clinically relevant biomarkers.
**Pre-analytical**	The laboratory validated in-house NGS workflows for specific specimen types (e.g., EBUS, fine-needle aspirates) used by other subspecialties.	Increased specimen adequacy and expanded access to rapid testing across sample types.
**Post-analytical**	Regular one-on-one meetings between laboratory and clinical stakeholders were used to review results, workflows, and issues.	Faster communication, improved alignment, and more timely clinical decision-making.

### Administrative burden

Administrative and reimbursement structures frequently lagged behind technical testing capability. MGH identified a high burden of compliance with federal and accreditation standards, often coupled with complex reimbursement negotiations and documentation requirements for novel tests. WOHS, while subject to provincial or regional regulatory structures, encountered its own administrative pressures, particularly around cost justification, funding approvals, and sustainability of routine use. Despite differences in the national healthcare environment, technical innovation must be accompanied by administrative alignment to ensure that rapid testing is not only feasible but sustainable.^46^ One key consideration for both sites was the integration across departments (“de-siloing”). Specifically, moving testing from clinician-initiated to reflex testing created a more tissue-oriented approach and reduced the need for front-line providers to play catch-up. As well, integrating NGS ordering within the existing system of pathologist ordering facilitated ease and speed of use. While these tests are implemented and performed in the laboratory, successful implementation requires interdepartmental collaboration across the institution.

### Implementation lessons and organizational considerations

Evaluation of the initial implementation phases at both sites highlighted key administrative and operational considerations. In both settings, stakeholders emphasized the importance of framing rapid testing initiatives around a business case that extended beyond clinical benefit alone. Implementation plans explicitly incorporated anticipated cost offsets, including fewer clinic visits and reduced rescheduling attributable to testing delays. Early engagement of medical oncologists and tissue-acquisition specialists (including radiology and surgical teams) helped align clinical priorities with operational and financial considerations, facilitating program adoption. Canadian data estimated that a patient with advanced NSCLC awaiting treatment initiation costs the healthcare system $400/day.^33^ NGS testing may also divert drug costs from payers to study sponsors as a result of patient enrollment in clinical trials.^47^^,^^48^ Comprehensive NGS is also more cost-effective than sequential testing.^49^^,^^50^

Additionally, significant risk of mortality exists during the interval between testing and results, with one study reporting that 6% of patients in their cohort had died prior to availability of the NGS report.^39^ Indeed, deteriorating health can be a major reason for not receiving matched therapies, requiring NGS-informed treatment selection earlier in a patient’s disease course.^51^ In patients for whom test results do not inform first‐line therapies, length of turnaround time is a frequent issue^52^ and oncologists report waiting longer than three weeks for results in some cases, resulting in treatment delays.^53^ A business case can therefore consider the costs of ineffective therapy, emergency care, delays, and inefficient workflows, while highlighting how innovation can improve care and support institutional investment. Accordingly, implementation planning frequently incorporated downstream healthcare utilization, workflow efficiency, and institutional resource allocation.

Clinical participation ensured that real-world bottlenecks and resource needs were captured and that the projected impact reflected actual practice patterns. Stakeholder buy-in was essential to identify workflow bottlenecks, establish operational ownership, and define institution-specific contingencies early during implementation. Participation of the pathologist and cancer care team in community outreach and relations can facilitate this process.^54^

Patient satisfaction, while valuable, was not an adequate standalone measure of program success. In the two contexts under study, clinician satisfaction was recommended as an easily obtained and objective performance indicator. Additional metrics such as the number of patients presenting for oncology consultation with complete biomarker data were also highly valued. The direct measurement of turnaround time remained a gold standard.

Implementation of streamlined and reflex molecular/NGS workflows in advanced NSCLC has been associated with shorter times from biopsy to treatment decision and initiation, reduced need for repeat biopsies through improved sample stewardship and increased use of liquid biopsy,^55^^,^^56^ and more efficient care pathways with fewer interim visits prior to treatment planning.^57^^,^^58^ Earlier initiation of appropriate targeted therapy is associated with lower reliance on unplanned acute care compared with some other systemic treatments, although direct evidence that rapid molecular testing alone reduces emergency department utilization remains limited.^59–62^

Briefly, we presented the results across multiple complementary levels. Figure 2 synthesizes a practical, stepwise approach to local implementation of rapid biomarker testing, illustrating how institutions can move from contextual assessment (Table 1), through evaluation of shared benefits and risks (Table 2), to identification of specific coordination and workflow elements that translate assay capability into patient-level impact (Table 3). Measurement of baseline and downstream clinical and operational outcomes can then provide the empirical basis for demonstrating the clinical and/or financial value of an integrated rapid biomarker testing strategy. Across both institutions and the reviewed literature, clinically actionable biomarker turnaround time emerged as a systems-level property dependent on coordinated rules, roles, relationships, and resources (Table 4).

**Figure 2.**
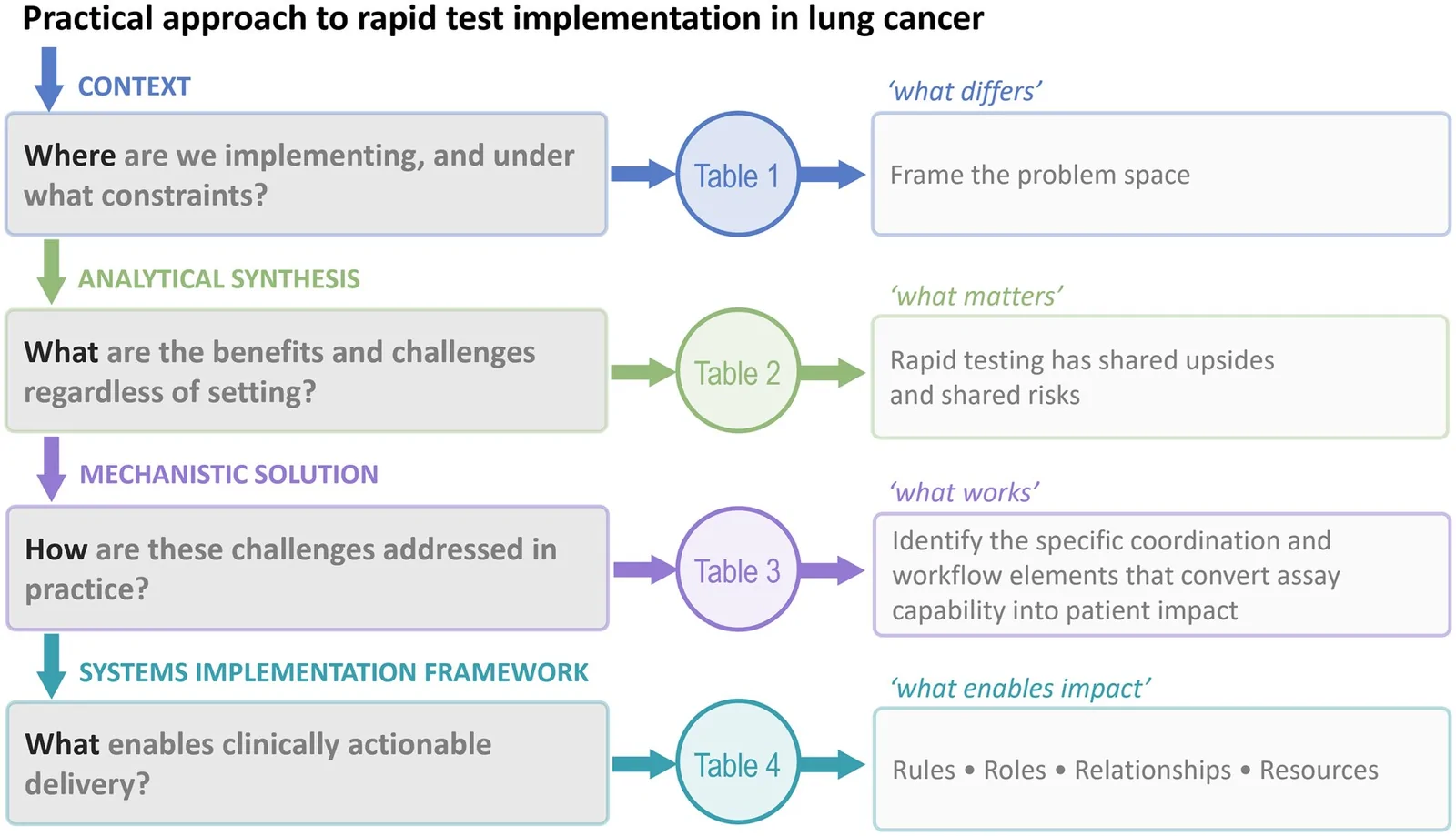
Practical approach to rapid biomarker testing implementation in lung cancer. This figure summarizes a systems-oriented implementation framework for rapid biomarker testing derived from the institutional experiences and literature synthesis presented in this study. The framework begins with contextual assessment of care settings, infrastructure, and operational constraints (Table 1; *“what differs”*), followed by analytical synthesis of the shared benefits, risks, and implementation pressures associated with rapid testing across environments (Table 2; *“what matters”*). The third level focuses on practical workflow and coordination strategies used to address these challenges in routine care (Table 3; *“what works”*). These elements culminate in a systems implementation framework identifying the operational rules, roles, relationships, and resources that collectively determine whether biomarker testing becomes clinically actionable within the therapeutic decision window (Table 4; *“what enables impact”*). Together, the framework emphasizes that the clinical utility of precision oncology depends not only on assay capability but also on coordinated workflow architecture and healthcare delivery systems.

**Table 4.** Systems implementation framework.

Systems principle	Operational domain	Example impact on turnaround time
**Rules**	Standardized procedural mechanisms	
	Reflex testing workflows	Reduces clinician-ordering delays
	Standardized ordering pathways	Enables immediate biomarker initiation
	Turnaround-time monitoring	Enables real-time identification of workflow bottlenecks
	Reimbursement and funding structures	Determines sustainability and scalability of rapid testing
	Coverage policies and testing authorization	May accelerate or delay biomarker initiation
**Roles**	Accountable actors	
	Interdepartmental coordination	Accelerates tissue triage and reporting
	Molecular pathology integration	Enables concurrent IHC/NGS workflows
	Dedicated molecular review and reporting	Improves rapid interpretation and clinical communication
**Relationships**	Coordination interfaces	
	Oncology–pathology communication	Aligns testing with therapeutic decision windows
	Tumor board integration	Facilitates coordinated treatment planning
**Resources**	Operational capacity	
**Infrastructure**	In-house versus send-out testing	Influences transport and batching delays
**Logistics**	Specimen routing and transit	Reduces pre-analytic latency
**Operations**	Workflow integration and automation	Enables parallelized processing
**Metrics**	Biomarker availability at first oncology visit	Measures clinically actionable delivery

Abbreviations: IHC, immunohistochemistry; NGS, next-generation sequencing.

## Discussion

Rapid molecular testing in NSCLC functions primarily as a systems implementation challenge rather than an isolated assay challenge. Its greatest value lies in accelerating treatment decisions, streamlining care pathways, and reducing diagnostic delays.^55–58^ From a systems perspective, rapid testing also supports more efficient resource allocation and offers a pathway to broader access by extending advanced diagnostics beyond specialized centers.^24^

Recent evidence provides broader context for the importance of these workflow considerations. In a large-scale analysis of comprehensive genomic profiling, the interval between biopsy and test ordering represented a major contributor to overall testing time, highlighting the importance of processes outside the analytical laboratory.^21^ Complementary modeling studies have estimated that incomplete or suboptimal biomarker testing may be associated with forgone survival and increased healthcare expenditures.^20^ Thus, realizing the clinical value of molecular testing requires effective implementation across the diagnostic and therapeutic pathway as a critical priority and the focus of this work.

Across these two settings, rapid testing in NSCLC showed a duality of promise and complexity that we organize into four complementary dimensions to guide implementation: *what differs*, *what matters*, *what works,* and *what enables impact* (Tables 1-4). While the overarching clinical goal (i.e., accelerating treatment decisions) remained constant, *what differs* between academic and community settings are the contextual drivers, constraints, and infrastructures that shape implementation strategies (Table 1). What matters across environments are shared benefits and challenges of rapid testing, including gains in timeliness balanced against analytical rigor, workflow integration, and reimbursement and regulatory contexts (Table 2). Operationally, what works are the concrete coordination mechanisms that align clinical, operational, and financial structures to enable timely, actionable testing (Table 3). Finally, what enables impact at the systems level are the operational rules, defined roles, interdepartmental relationships, and institutional resources that collectively determine whether biomarker testing becomes clinically actionable within the therapeutic decision window (Table 4). The case vignettes in Figure 1 illustrate the difference diagnostic and treatment trajectories can take in similar cases, with and without rapid testing. Together these elements are operationalized in Figure 2, which outlines how institutions can act when implementing rapid biomarker testing in routine care. In particular, outsourcing testing has been identified as a source of increased turnaround time in various studies.^31^^,^^63^ Although in-house testing is more likely to be available in a tertiary referral center,^63^ it is feasible to implement in a general hospital, as our analysis demonstrates. Barriers to implementation are context-dependent but include workload, workforce shortage, space issues, and reimbursement. Turnaround-time specifically is impacted by outsourcing, sample transport times,^31^ reflex compared to clinician-initiated testing,^64^ and batching requirements for NGS.^63^ Reflex testing emerged as a central operational requirement for rapid molecular testing programs, and given the overarching recommendations to perform molecular testing in all patients with NSCLC, it may even be considered as a rule for engagement. In our results, we believe that the two basic requirements for a rapid molecular testing program should include in-house testing availability and reflex testing strategies, which is borne out by other published literature,^8^^,^^31^^,^^37^^,^^44^^,^^64^ The availability of results at time of oncologist consultation is a vital metric and should be measured with accountability to quality in this regard as a critical element of cancer care delivery. Across both environments, successful implementation depended less on the availability of specific assays and more on the ability to re-engineer laboratory workflows, incorporating reflex testing, automation, and quality improvement principles. Clear interdepartmental communication and administrative alignment were essential to prevent gains in speed at one stage from being offset by constraints elsewhere in the care pathway. The systematic redesign of laboratory operations, coupled with competency assessment and turnaround time monitoring, ensured that rapid testing yielded measurable improvements rather than simply shifting bottlenecks. Simply put, speed is not an assay property, it is a system property.

Our findings also underscore that for academic centers, rapid testing is not a substitute for comprehensive molecular diagnostics but rather a complementary tool. Rapid assays provide early actionable results within clinically relevant timeframes^7^ and, in selected cases, may meaningfully influence the initial course of treatment.^65^ However, rapid testing is not intended to replace comprehensive molecular profiling in all patients or at all disease stages. Rather, broader sequencing is most appropriately reserved for situations where additional molecular detail is expected to inform management, for example at disease progression, in the setting of acquired resistance, or when first-line (rapid) testing is non-informative. This layered approach, deploying rapid testing to support timely first-line decisions while reserving more comprehensive profiling for defined clinical scenarios offers a pragmatic, value-based balance between speed and molecular depth. In community centers, resource constraints often preclude multiple parallel testing layers, underscoring the need for rapid testing strategies that achieve sufficient comprehensiveness to guide initial therapies. Integration with academic referral pathways can further extend this model by enabling access to advanced profiling when clinically indicated, without delaying upfront treatment. Several limitations apply. Our report focuses on NSCLC, a disease that often serves as a paradigm for precision medicine, and many of the principles described here extend to other tumor types with similar biomarker-driven treatment pathways. Our comparison centers on two successful implementations of rapid biomarker testing; unsuccessful or incomplete attempts (though equally informative) are rarely published and therefore not represented.

Our analysis focused on a setting in the Canadian healthcare context, as well as an academic center in the United States. Implementation took place within the broader healthcare environment, and adoption was influenced by external factors such as reimbursement schemes and the broader landscape of laboratory medicine. In both Canada and the United States, successful implementation depended on local institutional infrastructure, including diagnostic capabilities, clinical workflows, and multidisciplinary care pathways.^24^^,^^32^ However, the reimbursement environment differs substantially. In the Canadian setting, reimbursement for molecular testing follows provincial guidelines and is influenced in part by regulatory approval and drug coverage. Once funded, testing can generally be offered by any participating hospital, irrespective of its patient population. In the United States, reimbursement is payer-dependent, with coverage varying widely across public and private insurers.^9^ Our results are context dependent and underscore that implementation of rapid testing is beyond technical implementation alone,^46^ instead requiring a systems-based approach.

In addition, this work does not comprehensively address emerging complementary approaches, including digital pathology, AI-enabled multimodal diagnostics, circulating tumor DNA (ctDNA) testing, and minimal residual disease (MRD) monitoring, which may accelerate molecular insight and longitudinal treatment assessment but introduce distinct validation, governance, operational, and access considerations. As these technologies mature, their clinical impact will depend not only on analytical performance but also on successful integration into coordinated diagnostic and therapeutic workflows.

In summary, comparing two distinct health systems demonstrates that rapid testing can support equitable precision oncology when thoughtfully implemented. Together, academic innovation and community-based implementation provide a practical roadmap for broader adoption and consistent patient benefit.

## Data Availability

The data that support the findings of this study are available on request from the corresponding author.
